# How, when and why is emotional support delivered using videoconferencing by adult palliative care services successful? A realist synthesis

**DOI:** 10.1177/26323524251363271

**Published:** 2025-08-13

**Authors:** Michèle J. M. Wood, Amara Callistus Nwosu, David Dinham, Nicole Seneque, Catherine Walshe

**Affiliations:** 1 International Observatory on End of Life Care, Lancaster University, UK; 2 Marie Curie, London, UK; 3 Lancaster University Medical School, UK; 4 Marie Curie Hospice, Liverpool, UK; 5 Spectrum Centre for Mental Health, Lancaster University, UK

**Keywords:** realist review, videoconferencing, palliative care, emotional support, Normalisation Process Theory, telehealth, psychological care, art therapy, spiritual care, counselling

## Abstract

**Background:**

Videoconferencing (also known as telehealth) is part of digitally enabled healthcare provision (‘eHealth’) and its use in palliative care practice is increasing. There is uncertainty and limited evidence to guide organisations in how to use this technology to provide emotional support for patients, caregivers and the bereaved.

**Objectives:**

How, why, and in which circumstances can videoconferencing be used to successfully meet the emotional support needs of adults receiving palliative care?

**Design:**

Realist synthesis of literature was conducted according to RAMESES guidelines.

**Data sources and methods:**

Evidence of research studies and practice descriptions about successful emotional support interventions in palliative care by videoconferencing were identified from six databases (AMED, Medline, PsycINFO, SCOPUS, TRIP, Overton.io) and Google searching on16th January 2023. Normalisation Process Theory guided analysis and synthesis proceeded iteratively through retroductive reasoning.

**Results:**

Synthesis of 13 eligible sources (9 empirical studies and 4 practitioners’ perspectives) generated 10 context-mechanism-outcome configurations and 5 hypothetical explanations for successful videoconferencing interventions. Potential causative links were made connecting social isolation, financial, educational and relational resourcing, and feelings of self-confidence, fear, or desires for belonging.

**Conclusion:**

Emotional support by videoconferencing in adult palliative care is feasible when it addresses feelings of isolation and maintains patient/carer engagement with services. It depends on stakeholders being motivated and resourced to adapt and innovate interventions appropriate for those with least familiarity or access to technology. To be successful staff need leadership and organisational cultures that enhance their professional self-worth and technical competencies, that foster inter-agency collaborations and mitigate digital exclusion of service users.

## Plain language summary

How, when and why is emotional support delivered by video calls to adult patients, caregivers and the bereaved by palliative care organisations successful? A systematically conducted realist literature review

Connecting patients with healthcare providers by video technology (videoconferencing) claims to extend the reach of palliative care by overcoming barriers of geographical distance and limited time, energy and financial resources. Research in this area has focused on physical aspects of care. However, little is known about circumstances leading to successful emotional support by video call for palliative care service users provided by counsellors, arts therapists, chaplains, psychologists, social workers or medical and nursing professionals. To understand how, when and why videoconferencing is used successfully for emotional support in palliative care a systematically conducted review of literature was undertaken. Thirteen sources met the inclusion criteria and comprised research studies and practitioner accounts. These were appraised using Normalisation Process Theory, which offers ways to explain individual and collective efforts in getting new interventions underway in healthcare. A realist approach was used to analyse retrieved sources and build potential explanations to answer the review question. Five explanations were generated and labelled: 1/Shifting balance of power and agency; 2/All in this together; 3/Follow the leader or money; 4/Comply or die; 5/Fear of missing out. These suggest reasons for using videoconferencing for emotional support in palliative care. In circumstances of social isolation people seek new ways to stay connected with those who matter to them. With organisational support through the provision of material and educational resources patients and staff can adopt new interventions and be motivated to take account of folk with least familiarity or access to technology. Success requires leadership and organisational cultures that foster collaborations, professional development and technical competencies. This article may help organisations make best use of staff and technology to add virtual interventions to their palliative care provision.

## Introduction

Videoconferencing (also known as telehealth) is now part of digitally enabled healthcare provision (‘eHealth’) and involves using computer technology and the Internet to facilitate visual and verbal communication between users in different locations.^1,2^ Videoconferencing enables professionals, patients and their caregivers to simultaneously participate in virtual online meetings, and care can be agreed without delay.^3^ Recognised as time and cost-effective, videoconferencing has opened ways to dispense services flexibly and at scale.^4,5^ However, videoconferencing within palliative care was slow to take off and only accelerated during the COVID-19 pandemic, highlighting the benefits of videoconferencing for patients and their families towards the end of life.^6–10^ Despite the absence of in-person physical proximity videoconferencing can still enable meaningful therapeutic relationships between patients and professionals.^11,12^

Studies of the experiences of palliative care patients indicate that videoconferencing supports greater autonomy, better communication with professionals and promotes feelings of security.^13^ Caregivers find videoconferencing supportive through being included in care plans/meetings and being seen by professionals they feel freed of the burden to describe symptoms.^3,14,15^ As technology at all levels of the healthcare system – including in-patient and community palliative care – is being normalised, telehealth standards and guidance and survey instruments to evaluate telehealth encounters are being produced^16–18^ and the real-time interactions provided by videoconferencing show higher treatment adherence.^19^ This indicates an appetite for the implementation of telehealth within palliative care. However, issues of digital literacy, economic constraints and geographical limitations also influence implementation, and flag up the need for further research.

Much research on videoconferencing in palliative care has focused on physical aspects, yet evidence is emerging of its use for psychosocial interventions with this population; for example, Zoom (a popular videoconference software program) was used to facilitate online groups for families of critically-ill patients to address the emotional load of COVID-19 restrictions, or getting psychological care to patients with advanced cancer in resource-limited Mexico City.^20,21^ Prior to the COVID-19 pandemic there was a growing interest in hybrid, novel, synchronous and asynchronous digital psychosocial interventions for adults with life-shortening illnesses and their caregivers receiving palliative care, as detailed in a recent scoping review.^22^ While telehealth for palliative care service delivery has been prompted by COVID-19 mitigation measures, it is not yet clear *where* or *if* telehealth is extending the reach of palliative care, or *how* video-enabled interventions are utilised to *effect* successful outcomes.

The aim of the current review was to tackle these knowledge gaps by understanding the circumstances in which videoconferencing is used to deliver emotional support to adult palliative care patients and their families. By focusing on the affective rather than purely physical aspects of palliative care provision the intention of undertaking this review was to increase knowledge about video-enabled emotional support in palliative care. ‘Emotional support’ as an umbrella term was used to encompass psychosocial interventions such as arts therapies, counselling, chaplaincy, psychology and social work, and emotional interventions delivered by nursing and medical staff. Knowing why videoconferencing works well, and the factors that facilitate its success can help palliative care service providers target resources for maximum effect.

To understand this the review question posed was: how, why, and in which circumstances can videoconferencing be used to successfully meet the emotional support needs of adults receiving palliative care?

## Methods

To answer the review’s question a realist synthesis of literature was undertaken. Realist synthesis, also known as realist review, assumes that healthcare interventions (like all interpersonal activities) are complex interactions between participants’ characteristics, the contexts in which they occur and the interventions themselves. Understanding these relationships can help explain why the same interventions trigger different outcomes in different circumstances.^23,24^ Realist synthesis was considered most appropriate to understand which factors and circumstances trigger or inhibit the delivery of video-enabled emotional support in palliative care, resulting in successful outcomes. The theoretical framework for this synthesis was Normalisation Process Theory (NPT),^25^ which is an empirically derived explanatory model for implementation. NPT postulates how individual relationships and organisational processes enable complex interventions – such as telehealth – to become imbedded in healthcare.^26^ The concepts of NPT were used to identify relevant data sources and in the analytical process.

This paper is reported according to Realist And MEta-narrative Evidence Syntheses (RAMESES) guidelines.^27^

As a theory-driven approach realist synthesis goes below surface descriptions of what and how interventions work, to seek explanations for why certain behaviours and outcomes are observed in some circumstances and not in others. It does this by identifying potential causal relationships between contextual factors (context = C), the way these trigger people to respond (mechanisms = M), and the outcomes that result (outcome = O). In realist terminology, this is called ‘programme theory’ and represented as C + M = O. The objective of establishing likely explanations for the successful use of videoconferencing for emotional support in palliative care is to help service providers know when and in which circumstances to develop person-centred interventions that can be delivered remotely.

The synthesis process involved five discrete (albeit iterative) steps: clarifying the scope; searching for evidence; appraisal of sources to develop programme theory and data extraction; synthesis and theory refinement and dissemination of findings.^24^

### Step 1: Clarify scope

To identify potential explanations for successful palliative care emotional support by videoconferencing (hereafter referred to as ‘the programme’) a familiarisation search of relevant literature was undertaken, including searching with Publish or Perish software (https://harzing.com/resources/publish-or-perish). Papers from a scoping review that detailed digitally enabled psychosocial interventions with videoconferencing in palliative care^22^ provided initial thoughts about the reasons, resources and contexts that trigger emotional support outcomes. This helped identify ideas to formulate a rough/initial programme theory about the reasons for emotional support delivered in real-time at distance by videoconferencing (hereafter referred to as ‘video-enabled’ or ‘video call’).

### Step 2: Search

**Figure 1.**
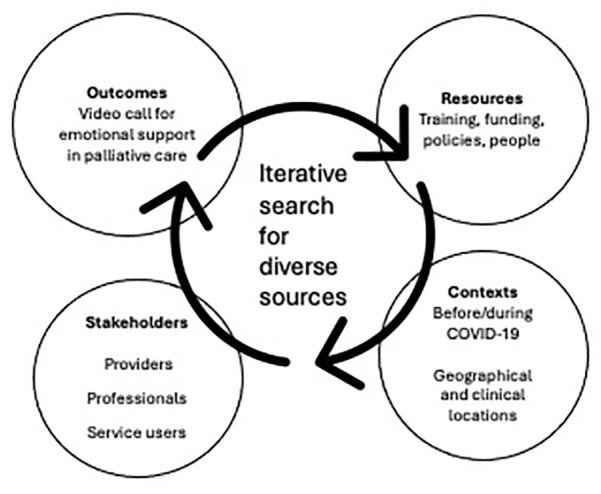
Iterative decision-making process for selecting sources.

Literature about emotional support interventions by video call in palliative care spans several knowledge domains so the following databases were searched: AMED, Medline, PsycINFO, SCOPUS, TRIP and Overton.io database. Websites were also searched for patient or carer accounts. Searching took place on 16th January 2023. There were no date restrictions. There were three search term concepts: videoconferencing; adult palliative care services and remote delivery of emotional, psychological, psychotherapy support interventions. Precise terms were adjusted as appropriate for each database. See Table 1 for eligibility criteria. Full search terms are presented in Supplemental Table A. Searching continued in an iterative manner as ideas became clearer during the review process; this is in keeping with the realist approach. Figure 1 shows the four foci that developed as searching proceeded. Citations were selected if they addressed or added different perspectives on outcomes, contexts, stakeholders and resources.

**Table 1.** Eligibility criteria.

Criteria	Include	Exclude
Types of papers	Empirical research any study design and non-empirical work (e.g. professions’ guidance documents, web pages, opinion pieces)	/
Language	English	Not English
Period	No date restrictions	/
Population	Adult (18+ years) patients with advanced life-shortening conditions; OR informal caregivers of adults receiving palliative care services; informal caregivers of adult patients with advanced life-shortening conditions; OR bereaved informal caregivers of adults receiving palliative care services	Patients and/or their informal caregivers with no diagnosed life-shortening conditions or terminal illnesses; children with palliative care needs
Intervention	Emotional support interventions delivered to adults using videoconference technology. These include (but are not limited to) interventions undertaken by healthcare practitioners and those trained in arts therapies, coaching, chaplaincy, counselling, psychology, psychotherapy, and mindfulness and delivered by any real-time video platform	Audio-only emotional support interventions such as telephone counselling, self-directed web interventions, mp3 files, apps for self-management
Context	Palliative care in community settings (own home, residential care or prison) or in-patients of specialist palliative care units	Remote monitoring or video consultations solely focused on physical symptoms or physical or medical regimens

### Step 3: Selection, appraisal and data extraction

Retrieved citations were imported to reference management software EndNote (https://endnote.com) and duplicates removed. Reference lists of systematic literature reviews found during the searching phase were scanned for further relevant papers. To facilitate teamwork all citations were imported to Rayyan (Systematic review software https://www.rayyan.ai/). Titles and abstracts were read by M.W., eliminating ineligible papers. A proportion of retrieved citations were double checked by three additional independent reviewers (C.W., A.N., N.S.), ensuring papers demonstrated sufficient information to address the review question. Thirty-two eligible papers were read in full; one was further excluded for the reason of double counting.^19^ Disagreements were resolved through discussion.

Retrieved sources were read to determine fitness for purpose noting their ‘relevance’ (does the paper contain data relevant to the topic or theories identified in step 1?); ‘rigour’ (is the source credible? Does it support the conclusions drawn from it?); ‘richness’ (how might this contribute to theory building or testing?)^28,29^ and their usefulness in contributing to the purpose of the synthesis. Supplemental Table B shows how this was operationalised for the retained sources. Papers deemed not relevant (*n* = 8) and maybe relevant (*n* = 9) were set aside. The remaining 15 citations for 13 papers were retained for appraisal.

Sources were scrutinised for the author’s explicit or implicit theory about why interventions work/succeed (or do not) and these data were extracted and assembled in a matrix (Supplemental Table B).

### Step 4: Analysis and synthesis

**Figure 2.**
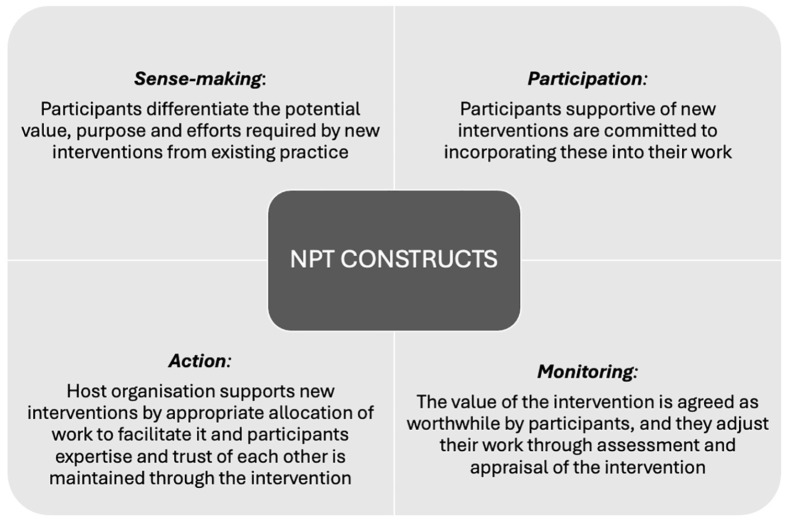
Four constructs of NPT. NPT: Normalisation Process Theory.

The four constructs of NPT^25^ are summarised in Figure 2.

These provided the review’s initial explanatory theory for how successful emotional support could be achieved by videoconferencing as set out in the following initial programme theory:

New video-enabled ways to deliver emotional support would be successful (*monitoring*) if staff and geographically isolated service users were committed to working together remotely (*sense*-*making*) because they valued seeing each other in real-time (*participation*), and they had the material resources and organisational support (*action*) to do this.

To test this, each included source was appraised using the NPT coding manual.^30^ The manual’s 12 questions aided data extraction, helping to target information about contexts, mechanisms, and outcomes of interventions. Ideas were generated for how each source addressed the review question. Synthesis developed through identifying the endogenous processes of relational work described between healthcare professionals and their patients, and exogenous processes that refer to the operational and institutional systems shaping how people act, both of which encompass NPT’s four constructs.^25^ Retroductive thinking aided by the heuristic ‘if-then-because’ helped identify potential motivators for the uptake of the programme by stakeholders. Synthesis was sense checked by a fourth independent reviewer (D.D.).

## Results

**Figure 3.**
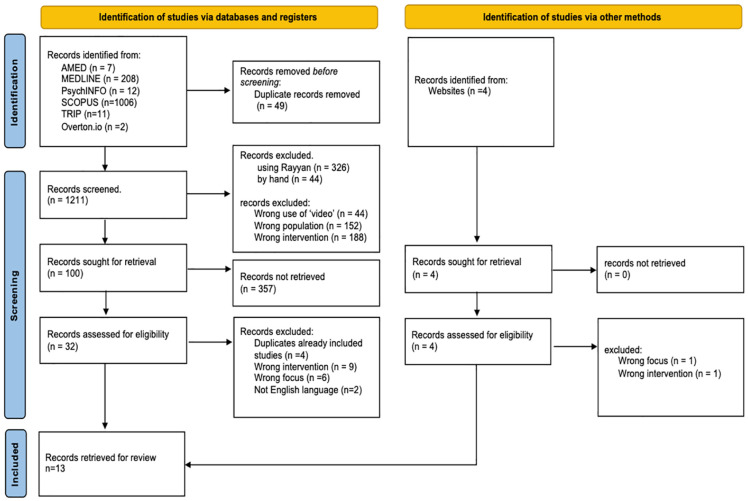
PRISMA flow diagram.

The search strategy resulting in the 13 sources synthesised in this review is shown in Figure 3. All English language publications, these sources were from North America (*n* = 7), United Kingdom (*n* = 4), Europe (*n* = 1) and South America (*n* = 1). They comprise nine empirical studies and four practitioners’ perspectives; key features of which are arrayed in Tables 2 and 3. Each source reports conditions for successfully implementing emotional support interventions (‘the programme’), where the programme was integral to existing palliative care provision^21,31–38^ or as a standalone innovative intervention for people with palliative care needs.^39–42^ The interventions span levels 1–4 of the UK’s NICE hierarchy of psychological support in palliative care^43^ ranging from nursing/medical professional empathic listening to specialist approaches delivered by psychologically trained practitioners embedded in multidisciplinary palliative care networks. All stakeholders had access to videoconference technology, and recipients of video-enabled interventions were unable to access usual in-person care due to their health condition or COVID-19 restrictions.

**Table 2.** Retrieved empirical sources 1–9 included in the review (*n* = 9).

First author (date); country	Setting and intervention	Research question	Design	Population	Findings
Stern (2012); Canada^31^	Community-based palliative care service telehealth 24-h intervention with videophone access to specialist nurses	To explore the perceptions of family caregivers and palliative cancer patients of home telehealth, and their experience with it	Mixed-methods case study Part of a randomised trial October 2004–April 2006	Patient/caregiver Dyads (*n* = 10) Bereaved family caregivers (*n* = 7)	Shows: Emotional support is integral to telehealth for palliative care; acceptability of video use and families’ experience video as reassuring Negative consequences of telehealth: A lack of integration of services, inappropriate timing of the intervention and technical problems
Middleton-Green (2019); England, UK^32^	Single point of contact with specialist nurse who could provide direct advice, referral on, and emotional or practical support	To evaluate ‘Gold Line’, a 24/7, nurse-led telephone and video consultation support service for patients thought to be in the last year of life	Review of data from electronic patient record on timing and nature of contacts with Gold Line plus qualitative evaluation of patients and carers	Patients (*n* = 8) family carers (*n* = 6) North of England Caseload review of patients (*n* = 1138)	The service predominantly accessed ‘out-of-hours’ for practical advice, symptom management, reassurance and emotional support was experienced as personalised, responsive, safe and efficient
Ozier (2019); Canada^39^	Remotely delivered intervention of 10 weekly structured cognitive and emotional therapy sessions	To develop and evaluate the feasibility of an innovative program to treat individuals with high-grade brain tumours via videoconferencing into participants’ homes to enhance accessibility	Feasibility study October 2015–May 2016	Patients with brain tumours (Grade III and IV) *N* = 5 Caucasian Canadians from British Columbia Cancer Agency centres	Video is acceptable platform for neuropsychological assessment, and for building therapeutic alliance
Milbury (2020); USA^40^	Four-session dyadic program integrating meditation training and emotional disclosure exercises delivered by FaceTime compared with usual care	To examine the feasibility and preliminary efficacy of a couple-based meditation program targeting symptom and wellbeing outcomes	Research pilot RCT	Patients with primary or metastatic brain tumours and their partners (*n* = 22) recruited with 77% non-Hispanic White	Videoconference delivery was acceptable Significant effects for patients Dyadic design increased feasibility and retention
Ritchey (2020); USA^33^	Veterans Affairs Medical Center Restructure of in-patient palliative care due to COVID-19 disruption	To identify the need for system innovation in palliative care, and a quality improvement approach to structure the project	Quality improvement project	71-Year-old man type 2 diabetes and COVID-19 pneumonia. (*n* = 1)	Yes Confirms pandemic as motivator for system change, with positive outcomes. But acknowledges that telehealth can ‘go wrong’ at the end of life
Chávarri-Guerra (2021); Mexico^21^	Adaptation of ‘Te Acompañamos’, a patient navigation program supported by MDT working remotely with patients between March 27 and June 15 2020	Service transformation	Qualitative online survey after receiving telemedicine interventions	Patients with advanced cancer (*n* = 45)	Describes conversion of in-person service to remote delivery because of COVID-19. Most common interventions were psychological care. Videoconferencing provided half of all interventions Identifies privacy issues, especially in the Mexican context of household overcrowding
Keenan (2021); Wales, UK^41^	Rurally based voluntary palliative care hospice at home service and community Specialist palliative care team at general hospital	What are the convergent and divergent views held by professionals and patients towards the implementation of telehealth in palliative care in relation to the principles of Self-Determination Theory?	Qualitative research with semi-structured interviews	Palliative care professionals (*n* = 8) Patients diagnosed with terminal cancer (*n* = 3)	Three themes identified that support other studies: Convergence and divergence of perceptions between patients and professionals explored using autonomy, competence, and relatedness. Differences highlighted
Palma (2021); Chile^35^	A hospital-centred spiritual and psychological palliative telehealth system during the pandemic, with a mobile palliative care team (physicians ×2, nurse ×1, psychologist ×1, and chaplain ×1) working across services in a 500-bed academic hospital	To describe the implementation of a spiritual and psychological palliative telehealth system during the pandemic	Pilot study May–September 2020	Hospitalised patients (*n* = 59)	High participant acceptance and user satisfaction. Videoconferencing can deliver spiritual care
Pearce (2021); Ireland^36^	Range of bereavement care provision: including emotional support and listening, and specialist psychology and psychiatric support	To investigate the experiences and views of practitioners in the UK and Ireland concerning changes in bereavement care during the COVID-19 pandemic	Research August–September 2020 UK and Ireland Online survey using snowballing sampling	Health and social care professionals involved in bereavement support from 13 geographical locations (*n* = 805)	90% reported changes to bereavement care practice through use of telephone, video, and other forms of remote support Findings challenge ‘positive’ narrative about virtual remote support for families

MDT: Multidisciplinary Team.

**Table 3.** Retrieved non-empirical sources 10–13 included in the review (*n* = 4).

First author (date); country	Source type	Source setting and purpose	Population	Key takeaway
Calton (2020); USA^34^	Opinion piece	Gives telemedicine quick tips based on authors and colleagues experience at UCSF and Resolution Care Network	For out-patient population but suggests applicable to in-patients too	Authors describe the relational efforts required to enable successful video consultations and health system level changes to fund these. Significance of emotional communication online acknowledged
Hospice UK (2020); England, UK^37^	Website blog	Highlights hospice care during COVID-19 when consultations done by phone or video link. Access to the ward was limited; Staff from social work team and spiritual care team arranged video meets with patient which he really appreciated	Family caregiver’s story of hospice care for her husband during COVID-19 pandemic. He had heart failure (*n* = 1)	Videoconferencing successfully folded into usual care by attentive staff enabled emotional support for patient and wife bridging out-patient and in-patient experiences. Illustrates hospice care is for people of colour, of different faiths, with non-cancer diagnoses, and families
Hospice UK (2022); England, UK^38^	Sector consultation document	Submission to Government’s 10-Year Plan for Mental Health and Wellbeing in England. Flags up needs of people across the lifespan living with terminal or life-limiting conditions and their families and friends, and the hospice workforce	National Hospice sector	Identifies benefits and challenges of online services Provides contextual information linking mental health, impact of health inequalities, digital literacy for staff and patients, and economic constraints on service development
Mackey (2022); USA^42^	Clinical practice report	Case description of Intensive Care Unit Palliative care consult for symptom management and goals of care for 73-year-old man	Palliative care patient (*n* = 1)	Illustrates multidisciplinary team care during COVID-19 and how technology was used to deliver emotional and spiritual care. Makes the point that the advantages may only be limited to those with skills and technical resources

UCSF: University of California, San Francisco.

### Step 5: Main findings

This realist synthesis tested NPT’s assumptions about the relational work done by those delivering and receiving video-enabled emotional support in palliative care as set out in the initial programme theory. It generated hypothetical causal relationships between such interventions’ contexts, mechanism and successful outcomes. The findings are reported here in narrative form to illustrate these potential causative links and to highlight connections with NPT’s four constructs. Ten context-mechanism-outcome configurations (CMCOs) were developed from which five rival and sibling explanations were postulated to explain how, when and why video-enabled emotional support maybe successfully implemented. These five explanations address the efforts made by people (individually and collectively) to adopt a new intervention. They represent hypotheses to test NPT’s framing of the programme theory set out at the start and were refined further through consideration of how the COVID-19 pandemic changed access to services and funding. These are set out below, include the NPT descriptions and linked to relevant CMOCS, along with illustrative quotes.

### ‘Shifting balance of power and agency’ (CMOCs 1–3)

Taking up video-enabled emotional support interventions in contexts of service innovation or circumstances of a global pandemic inevitably shifts how people interact with each other. Whether patients and carers receiving emotional support video calls at home^32,39,41^ or in the in-patient unit,^33^ more effort is required by staff to ensure the programme works and to collaborate with the recipients of their care.^35^ This is potentially an increased burden on staff, triggering reluctance to adopt this way of working. The relational activities in planning and resourcing videoconferencing, along with carrying out the intervention necessitates all stakeholders to agree on its value. For patients and carers, the benefits are the conservation of financial and physical resources usually expended on going to healthcare meetings in the hospice or hospital site. Staying home also means patients feel less dependent on others to transport them to appointments. Instead, the healthcare professional becomes a guest in the patient’s home, and the patient, being more relaxed, feels empowered to engage actively in the video call. People to advocate/support the patient can also be added virtually to the meeting. The efforts made to implement the programme shifts the power dynamic in favour of the patient or carer:

I think sometimes when you go face-to-face, especially with some of the big consultants, it’s kind of a bit daunting or a bit off putting or they’re kind of a bit, like, okay I’ve got so much time there’s a whole waiting room of people out there. Whereas if you’re on telehealth maybe it would give you a bit more confidence to say, oh wait a minute I need to just ask you this or hang on I need to ask you this. And just give you that bit more confidence because you’re not actually there with them. . . you’re in their space aren’t you, whereas at home you’re on your own grounds, so you’re kind of more confident anyway. ‘Patricia, Time point 2, 202’ P4 Keenan et al.^41^

For healthcare professionals the change in working practice also conserves time travelling to see patients at home, especially those in rural locations:

Many clinicians note telemedicine visits are shorter and more focused than in-person visits. While this can increase efficiency, creating space for patients and families to share their thoughts and feelings is arguably even more important than it was before the COVID-19 pandemic. e13 Calton et al.^34^

When circumstances provide staff with new opportunities to innovate services that connect with new patient groups, shifts in ways of working can benefit all stakeholders. For instance, Ozier et al. found that a structured 10-weekly counselling intervention by video call worked well for people with challenging symptoms of cognitive impairment due to a brain tumour.^39^ However, shifts in usual professional-patient relationships can leave staff feeling less than satisfied with their work, especially where non-verbal interpersonal exchanges usually core to their roles are removed by using videoconferencing:

The lack of human touch was identified as a barrier for professionals as they feared that patients would consider the care provided using telehealth to be impersonal. p7 Keenan et al.^41^

Context-mechanism-outcome configurations:

1. The provision of the programme (emotional support by videoconferencing) [C] empowers patients [M] to engage more actively with professionals [O] because they feel more comfortable in their own home [M] [C].
2. When circumstances provide new opportunities for staff to innovate services [C] staff feelings of self-efficacy and job satisfaction increase [M] as they provide virtual interventions to deliver emotional support interventions [O].
3. Implementing the video-enabled emotional support intervention [C] disempowers staff who feel a loss of being able to deliver the human touch that is core to their practice [M] and job satisfaction decreases [O].

### ‘All in this together’ (CMOCs 4–5)

In the context of a crisis that prohibits usual in-person care, creating networks of participation around a new practice, and finding ways for everyone to work together are the social mechanisms proposed by NPT for successful implementation. In such situations patients and professionals are motivated by a desire to receive or deliver a service:

Throughout the lockdown we felt supported. Anne, from the social work team called regularly, sometimes just to see how I was, and the spiritual care team have been wonderful. Laurence from the team arranged video meets with Sunil which he really appreciated and he calls me too. Hospice UK^37^

Staff work together to make the transition from in-person to online therapeutic encounters accessible for service users. They build into the virtual environment the same kinds of values (or social affordances) that are qualities of the in-person setting.^34,35,41^ This may require delivering materials resources, technical support, online communication etiquette and staff training to everyone involved:

The PCT nurse coordinator (A.F.) arranged for the tablet to be brought to the patient’s room, scheduled times for each call, and obtained contact information for family members. The coordinator prepared the family regarding the patient’s condition and what they might see in the ICU room. The chaplain, ICU nurse practitioner, and bedside nurse donned PPE and established a video link with each family member using the tablet. The chaplain sang hymns, read his favorite Bible verses, and prayed with them. P995 Ritchey et al.^33^

This has been a difficult time for both the bereaved and staff. The bereaved have a reduced, non face to face service. The staff feel powerless and are restricted from doing the job they are passionate about. That said a great deal of learning has been going on and staff have been imaginative in finding new approaches. ‘(#418 palliative care specialist nurse)’ p7 Pearce et al.^36^

Context-mechanism-outcome configurations:

4. Isolation due to the closure of in-person services prevents care as usual [C] so staff and patients learn new skills together [M] to adopt the programme. This builds reciprocity and despite absent physical proximity the therapeutic alliance is strengthened [O].
5. A terminal diagnosis halts a person’s usual life and activities [C] hospice staff work together with patient and family building relationships [M] that bridge in-patient and home settings facilitated by videoconferencing [O].

### ‘Fear of missing out’ (CMOCs 6–7)

Another explanation for outcomes in circumstances where ‘care as usual’ is no longer possible – such as during COVID-19 restrictions – is people’s fear of missing out or of being excluded, which could result if they do not transition to video call interventions. For the professional this may be a fear of not being able to do their job, resonating with ‘shifting balance of power and agency’, or anxiety that ceasing to work as usual diminishes their professional identity and worth. For example, when new bereavement services developed during COVID-19, others were suspended, and staff were furloughed. Such situations mobilise stakeholders to see the benefits of taking up new practices, in NPT terms this is ‘sense-making’ (see Figure 2)

The pandemic was a powerful motivator for systemic change that propelled us to find new ways to provide caring and connectedness for patients at the EOL and their families. Our experience shows that although technology does not replace face-to-face encounters, it can offer meaningful connection. P996 Ritchey et al.^33^

The social isolation required during the pandemic compounded patient and family stressors and diminished the patient’s access to clinicians and to his usual support network and coping strategies. p203 Mackey et al.^42^

I feel it’s the isolation that is causing the greatest emotional and mental anguish. p7 Pearce et al.^36^

For the patient, accepting the programme may be motivated by a fear of isolation, or concerns about missing out on important relationships or receiving services.

Mr. H enjoyed his virtual visits with the palliative care team, reporting that it gave him comfort to see team members’ faces, even if not in person. p204 Mackey et al.^42^

Context-mechanism-outcome configurations:

6. With limited or no access to in-person emotional support interventions [C] fear of being isolated motivates staff and patients to engage differently to work together [M] ensuring they implement the programme [O].
7. Maintaining or developing an online relationship through videoconferencing [C] helps isolated people feel they are part of a validating community [M] and being an important part of a network, the video intervention is deemed successful [O].

### ‘Follow the leader/money’ (CMOCs 8–9)

Change is made possible when institutional level practices organise new ways in which people work to implement the programme. NPT describes these as ‘implementation contexts’. These make up an organisation’s resources, providing structure and procedures for the settings in which the new intervention can be operationalised. This includes social relations where those with high status, power, or control of finances unlock these resources, in NPT terms this is ‘action’. Staff are thereby incentivised and motivated to adopt new practices:

. . .the University of California, San Francisco (UCSF) has mandated telemedicine be used to care for palliative care and nonpalliative care patients in ambulatory settings, whenever possible. Similarly, many hospice agencies are currently offering most, if not all, social work and chaplaincy support by telemedicine. [. . .]. To support these changes, many telemedicine regulatory measures are being relaxed. e12 Calton et al.^34^

In times of systemic disruption, innovation is required to overcome barriers and identify new strategies for a business to persevere (e.g. maintain healthcare service delivery). [. . .]

The prerequisites to innovation include not only knowledge, expertise and experience, but also courage, creativity and fearless leadership. p993 Ritchey et al.^33^

Where political mandates determine organisational funding, leadership of the knowledge specialist can be an advantage.

The Gold Line offers, in the minds of our participants, the opportunity to elude the structural barriers to accessing responsive care by enabling faster responses by GPs, District nurses and accident and emergency departments because a ‘specialist’ service has enabled it. p5 Middleton-Green et al.^32^

Context-mechanism-outcome configuration:

8. Usual working practices halted by COVID-19 restrictions challenge employers to review policies and procedures[C]. Service leaders feel anxious [M] to maintain current commitments to patients and funders, so staff roles, policies and protocols are updated [M] to enable remote delivery of emotional support interventions [O].
9. Where leaders release knowledge and material resources for staff to work differently [C] staff and patients are inspired or incentivised to follow [M] with the outcome that the programme succeeds [O].

### ‘Comply or die’ (CMOC 10)

Pressure exerted within organisations to change working practices may be triggered by financial and job losses. People worried about employment security may feel pressured to take up new ways of video-enabled working, which reduces rather than maintains usual in-person emotional support. This illustrates NPT’s sense-making construct. This explanation contrasts with the positive views described above; the implementation of the programme constrains services:

Bereavement care fell to a wide range of staff members, including some with limited experience of or training in supporting bereaved people who had to rapidly develop the required communication skills. p3 Pearce et al.^36^

In 2020, COVID-19 restrictions and donor confidence prompted a funding crisis in the hospice sector, resulting in a 40% drop in hospice fundraising in 2020–2021. Government stepped in with emergency funding to purchase capacity from the sector, without which many hospices would have struggled to survive. However, this most welcome emergency funding has now ended and, without a sustainable funding model, hospices struggle to raise enough money to provide services that are vital to the mental health and wellbeing of their patients. p2 Hospice UK^38^

Context-mechanism-outcome configuration:

10. In circumstances of financial constraints [C] service providers review resources and being fearful of extinction implement virtual services delivered by videoconferencing [M] as an opportunity to ‘do more with less’ [O].

### Refining programme theory

These five explanations address ways people may work individually and collectively to adopt a new intervention. These can occur across the healthcare ecosystem and are not mutually exclusive. In fact, a cascading influence is suggested that show how generative mechanisms move from organisational leader to health professional to service user, as Pawson states ‘The actual intervention takes shape according to the power of the respective parties’ p3 (24).

The findings suggest that implementing the programme changes power dynamics between patients and practitioners activating several causal chains of behaviour, that are responsive to financial and interpersonal contexts.

A significant trigger for change was the COVID-19 pandemic. Prior to this, sources report that uses of videoconferencing for palliative care delivery was slow despite staff readiness and patient acceptability.^31,33^

As the pandemic unfolded, there was a general embrace of rapid initiation and utilization of telehealth by the PCT [palliative care team], patients, and the entire healthcare system. p994 Ritchey et al.^33^

With increased self-confidence mobilised by engaging in videoconferencing it seems that patients and caregivers may value taking more ownership of their healthcare consultations. Where health professionals feel motivated to collaborate with patients through video-enabled interventions both may gain an increased sense of competency and self-worth. Consequently, if the uptake of the programme occurs in a relational context that alleviates fear and isolation positive therapeutic alliances can be established and a successful outcome achieved.^37^ This point is reinforced by Sarmento et al. whose research highlights the importance of reducing fear to engender feelings of security in palliative patients being cared for at home.^44^

While positive outcomes may result from actions by well-resourced stakeholders responding to circumstances where life as usual is disrupted, this may not always be the case. In fact, getting access to palliative care is difficult for those in financial hardship, which itself is a cause of social isolation.^45^ Videoconferencing may be regarded as a good way to address social isolation, but only having options of emotional support by videoconferencing may reinforce feelings of exclusion and isolation from palliative care services.^38^ Services cannot rely on videoconferencing as a quick fix for social exclusion. The interwoven factors of deprivation faced by those with palliative care needs (including limited material resources, technical knowledge and skills), must be addressed first. The collective efforts to address educational and material limitations outlined in the included papers (and hypothesised in the synthesis) suggest ways mitigation can be made to prevent exclusion.

Financial resources, staff digital literacy and enthusiasm for adopting a new way of working influence the long-term sustainability of video-enabled emotional support once COVID-19 is controlled. The normalisation of the new intervention was picked up during data analysis by question 12 of the NPT framework ‘How have interventions and their components become incorporated in practice?’ Reported interventions offer templates for safe management of patient care during COVD-19 or potential future service developments rather than foundations that are being built on. Pearce and others warn that reduced income for palliative care – delivered mainly by charities – will negatively impact the provision of care.^36,38^ In the context of financial scarcity an organisation’s viability may be threatened, leading to choices to redeploy staff into new roles. If these staff feel neither trained or committed to this, feelings of being deskilled and demotivated can result.^46^

The initial programme theory was refined through the synthesis process as:

Successful emotional support delivered by videoconferencing in palliative care is feasible when it addresses feelings of isolation and maintains patient/carer engagement with services. It depends on stakeholders being motivated and resourced to adapt and innovate interventions appropriate for those least familiar with technology. To do this staff need leadership and organisational cultures that enhance their professional self-worth and technical competencies, that foster inter-agency collaborations and mitigate digital exclusion of service users.

This refined programme theory supports NPT’s four core constructs (sense-making, participation, action and reflexive monitoring) by recognising how networks of relationships between patients, professionals and organisational managers can facilitate changes in practice across the healthcare ecosystem. It suggests that collaborative relationships are most likely to be activated in circumstances characterised by social isolation or where people feel in danger of alienation from what is important to them. As a social action theory focused on collective efforts to implement and sustain new interventions NPT does not account for feelings implied by people’s responses to the opportunity of video-enabled emotional support interventions. Further theories were sought.

## Discussion

This realist synthesis of 13 sources established five likely explanations to answer the review question of how, why and in which circumstances emotional support by videoconferencing is successfully adopted. Two semi-predictable patterns (demi-regularities) were identified across the papers to explain motivators for stakeholder efforts resulting in successful outcomes. These were how people in leadership responded to COVID-19 through changes in resource allocation (in NPT this is an exogenous process), and ways geographically isolated stakeholders formed networks of support to remain connected (NPT’s endogenous process). Both support NPT’s constructs.

Understanding why leadership and stakeholder networks achieved successful outcomes 10 context-mechanism-outcome configurations were theorised. Leadership that released financial/material resources as a stimulus for action, could be both context and mechanism, prompting stakeholders to feel valued, and reinforcing a view that engaging with the programme has high status (‘All in this together’ and ‘Follow the leader/money’). Without positive leadership and with limited collective ‘buy-in’ from stakeholders simply releasing resources may trigger feelings of pressure, as described by ‘Fear of missing out’ and ‘Comply or die’. Both ‘Shifting balance of power and agency’ and ‘All in this together’ suggest successful adoption of videoconferencing by patients and carers might rest on a change in the power differential between expert (professional) and novice (patient). Where staff are willing to adjust usual practices to accommodate videoconferencing the action of learning together provides a helpful shared task around which teams and patients may bond, thereby creating or strengthening stakeholder networks of trust.

Notably, this synthesis did not posit ‘for whom?’, a query often included in a realist review. The boundary set for the current review question was videoconferencing usage in palliative care delivery; by implication all stakeholders had access to devices and internet data. Unsurprisingly, literature came from upper-middle income and high-income group countries. Given this, selection of sources was made looking for diversity of features known to be both a benefit and barrier to the implementation of telehealth for palliative care. Efforts reported in the included papers illustrate mechanisms to overcome potential digital health inequalities when people are motivated to address them. For instance, disparities in the digital literacy of stakeholders were tackled through education activities.^35,41^ Economic constraints of service users were overcome through the provision of equipment or by developing interventions that match the functionality of devices that most people had. Chávarri-Guerra et al. point out that despite limited incomes of most Mexican households, a large percentage had an internet connection and more that 90% possessed a smartphone, the device most people used to receive their intervention. However, the authors note household overcrowding in the Mexican context meant confidentiality was an issue.^21^

Digital exclusion through factors of financial hardship, digital literacy, geographical and political constraints, gender, age and individual preference have been widely reported as structural inequities of telehealth.^47,48^ This is supported by Mackey et al. who notes the advantages of telehealth may only be limited to those with skills and technical resources.^42^ However, attention needs to be given to staff and service users with lower educational attainment, less management experience, those living on lower household incomes, and from minoritised ethnicities as there is evidence that people in these groups are least likely to engage with remote consultations.^49,50^ While ‘scarcity’ may trigger actions towards successful technological innovations and collective willingness to act together,^10,51^ without relational and material resources structural inequalities will persist. Leadership cultures in different health systems around the world may vary, but outcomes will still depend on who has most power to release or withhold the resources that drive change.

The outcomes of this review raise questions about where the financial burdens and benefits fall when implementing videoconferencing and other digital tools in palliative care.^36,38^ While several studies claim lower cost benefits of telehealth for the patient^4,52^ isolating the economic value of digital psychosocial interventions integral to palliative care services is complex. Little is known about the economics of digital health interventions in particular. Three recent systematic reviews outline the challenges of establishing whether the palliative care approach in general is cost-effective.^53–55^ They identify challenges with choice of appropriate measures, reliability of cost information, and the limited range of perspectives sought.

Outside palliative care, reviews of the economics of digital health interventions have shown them to be cost efficient or cost neutral, notwithstanding similar methodological limitations. In systematically reviewing digital health interventions for people with heart failure Zakiyah et al. found these were more cost-effective than non-digital options.^56^ In a study comparing in-person with videoconference-delivered cognitive behavioural therapy in mental health researchers found that video was slightly more expensive for provider institutions but less burdensome for patients and concluded that videoconference delivery was cost-effective.^57^

For those seeking to commission or develop cost-effective digitally enabled palliative care services navigating the economic evaluation research literature is complicated. Fischer et al. recommend paying close attention to the context-specific features of any economic evaluation of palliative care services.^55^ With the normalisation of videoconferencing for healthcare delivery, researchers would do well to include service users’ valuation of their resources and societal perspectives in future economic evaluations.

Findings from this synthesis highlight the importance of first understanding and addressing barriers to palliative care per se before routinely implementing videoconferencing.

### NPT and alternative theory development from CMOCs

As outlined already NPT and realist synthesis were used to answer the review question. While NPT is increasingly aligning with a realist approach, it is conceptually different to it.^58^ NPT puts its focus on actions taken through relational work which are shaped by organisational structures. Realism uses abductive and retroductive reasoning to consider hidden motivators and contextual determinants driving these actions.^23^ In realist terms, feelings are understood to activate behaviours in response to contextual conditions, and this synthesis highlighted how feelings of isolation in situations of living with terminal illness, or during COVID-19 pandemic restrictions or disrupted employment conditions may mobilise people to engage with videoconferencing if given the opportunity. NPT explained ‘how’ success is mobilised through the collective and individual actions of people working together. However, while NPT does describe ‘mechanisms’ in its own terms^30^ it was clear in the synthesis process that NPT could not fully account for people’s motivation and feelings (the why), or their responses to available resources (what) which could lead them to behave as they did.^58^ Three alternative theories were considered to see if they better addressed this.

1. Connectivism, a learning theory developed to explain adult learning in the online environment^59^ might explain what educational resources are required to motivate staff and service users to use videoconferencing. Education about technology as an enabling factor for successful use of videoconferencing was evident as a facilitating mechanism in this synthesis and is supported in the wider literature.^60^
2. Self-determination theory, a psychological theory of motivation that values personal autonomy, competence and relatedness explains changes on a personal level. This reinforces the positive experiences of the programme for individual service users and staff.^61^
3. ‘Belongingness’ is the motivation people have to seek affiliations with others, enabling them to develop and maintain a sense of self-worth.^62^ The need to belong motivates companionship, interpersonal connections, and affiliations, and is built through reciprocal emotion-sharing and proximity to others across the lifespan. This explanation encompasses individual and collective perspectives, bringing together psychological, evolutionary, and sociological theories. Belongingness is also a cognitive process where similarities of self and others determine self-worth and membership of the group. To avoid exclusion/isolation and to gain a better chance of belonging, people present themselves in ways that are socially approved of.

Belongingness raises awareness of the value of social connectedness for wellbeing^63^ and as a mitigation for loneliness. Expanding NPT’s explanation for the relationships generated in this synthesis by adding in other theories (‘layering theories’)^64^ is an outcome of this synthesis. This may explain the different levels in the programme theory, where implementing the emotional support intervention (itself a complex system) may benefit some stakeholders while disadvantaging others. The value of belongingness for a palliative care population is an important outcome of this review and supports Bradley’s research on hospice day service.^65^

### Strengths, limitations and future research

A strength of this realist synthesis is its focus on emotional support interventions and the factors that combine to make these possible using videoconferencing in palliative care. Understanding why, when and how to deliver such interventions successfully could be valuable to providers and service users wanting to use technology to extend the reach of in-person services. This review recognises that emotional support is integral to the care of those living with non-curative illnesses and deserves increased investigation.^66^ Understanding the different cultural contexts for emotional support interventions was beyond the scope of this review, however NPT’s emphasis on building trust through collective efforts is of relevance.

This review had several limitations. Only using English language publications narrowed the pool of sources, excluding other healthcare systems and cultures where emotional support interventions and usages of videoconferencing may be different from sources included here. Two recent systematic reviews highlight different structural inequities of digital health policies across the socio-demographic spectrum. For people living in low- and middle-income countries technological and social infrastructure problems are significant barriers to the reliable implementation of digital health interventions.^67^ In upper-middle income and high-income group countries like those of our included sources people least likely to access health services by videoconferencing were aged over 65 years, had lower household incomes and lower educational attainment and English was not their first language.^47^ The depth of data within the included studies meant it was not possible to conduct a comparative analysis across different healthcare systems, although we do acknowledge that culture and context will have a major effect on implementation. We have noted this in areas where it seemed particularly important. Efforts to build trust and a sense of belonging central to the programme theory developed in this synthesis align well with the cultural safety that Moecke et al. argue is critical for culturally-informed telehealth services for indigenous groups in United States, New Zealand, Canada and Australia.^68^

Another constraint noted in searching the literature is the under-reporting of psychological practitioners’ contributions to the multi-professional care of patients and families. To overcome this ‘emotional support’, initially defined using UK NICE guidance^43,69^ as embracing interventions by psychological specialists at levels 3–4, was widened to include interventions by health and social care professionals at levels 1–2. While a systematic approach was taken to this review it is possible that synthesis of other sources not included here may provide different explanations.

This review considered literature arising from two distinct historical times (before and during COVID-19), where the pandemic shifted videoconferencing from novel interventions (context) to essential activities (mechanisms). Videoconferencing normalised during COVID-19 has become mainstream and is increasingly used to mitigate the pandemic’s unfolding effects on the mental wellbeing of the general population. Whether palliative care providers continue to adapt their services to include videoconferencing will depend on how they have been impacted by factors described in this review.^70^ Sustainability of telehealth interventions as an integrated part of palliative care will depend on different factors, including concerns that if videoconferencing replaces in-person sessions the quality of interventions will suffer.^36^ This realist synthesis highlights how intrapersonal, interpersonal and structural features are variously activated by the contexts in which palliative care is delivered.

### Recommendations

One recommendation from this review is for better training of practitioners to confidently deliver online interventions, and to assess and meet service users’ psychological needs. This is consistent with Levoy et al.’s rapid review of palliative care delivery changes during the pandemic which found the increased demand for psychological and spiritual support was not adequately met by existing services.^71^ Likewise, a recent survey of hospice care professionals felt they lacked sufficient knowledge and skills to address psychological issues.^72^ Finally, consistent documentation of where in the patient and service care pathway emotional or psychological support is identified and delivered would improve clarity and could improve overall care.^73^ Further research on specific system-level factors and stakeholder roles (e.g. healthcare professionals, caregivers, patients, and policymakers) that facilitate or hinder implementation of videoconferencing for emotional support would be a valuable next step.

## Conclusion

Successful emotional support by videoconferencing in palliative care is feasible in circumstances where technical infrastructure facilitates remote healthcare services, and where stakeholders have established sufficient interpersonal trust. Then emotional support by video call interventions can address feelings of isolation and maintain patient/carer engagement with services. It depends on stakeholders being motivated and resourced to adapt and innovate interventions appropriate for those with least familiarity or access to technology. To be successful staff need leadership and organisational cultures that enhance their professional self-worth and technical competencies, that foster inter-agency collaborations and the positive feelings of belonging that this way of connecting at distance can bring.

## Supplemental Material

sj-docx-1-pcr-10.1177_26323524251363271 – Supplemental material for How, when and why is emotional support delivered using videoconferencing by adult palliative care services successful? A realist synthesis Supplemental material, sj-docx-1-pcr-10.1177_26323524251363271 for How, when and why is emotional support delivered using videoconferencing by adult palliative care services successful? A realist synthesis by Michèle J. M. Wood, Amara Callistus Nwosu, David Dinham, Nicole Seneque and Catherine Walshe in Palliative Care and Social Practice
