# Supplementary material for: How, when and why is emotional support delivered using videoconferencing by adult palliative care services successful? A realist synthesis
Source: Palliat Care Soc Pract. 2025 Aug 13;19:26323524251363271. doi: 10.1177/26323524251363271 (PMC12357020; doi:10.1177/26323524251363271)
Supplement: sj-docx-1-pcr-10.1177_26323524251363271 – Supplemental material for How, when and why is emotional support delivered using videoconferencing by adult palliative care services successful? A realist synthesis [file sj-docx-1-pcr-10.1177_26323524251363271.docx]

**Table A Search terms**

| CONCEPT | Search terms/ Key words/ MeSH |
| --- | --- |
| 1. videoconferencing technology | Teleconferenc* Telemedicine Telehealth Telehospice Videoconferenc* Teleconsult* Telepractice Telepsych* Telerehab* "Telepsychiatry" OR "Telepsychology" virtual KW: Tele health, videoconferencing, virtual services, or interventions; MeSH: Videoconferencing, Telemedicine, Remote consultation |
| 1. Adult patients and informal caregivers using palliative care services | Hospice Palliative advanced care terminally ill KW: Palliative care, adults receiving palliative care services as patients, carers or bereaved; MeSH: Palliative care, Terminal care, Hospice and palliative care nursing, Palliative medicine |
| 1. Remote delivery of emotional, psychological, psychotherapy support interventions | “online therapy” Counsel* "Computer Assisted Therapy" Psychotherapy+ KW: psychosocial care, emotional support, ambulatory care psychology, Counseling methods; Professional-Patient Relations; Psychotherapy methods MeSH: Psychosocial Support Systems, Emotional adjustment, Psychosocial intervention, Psycho-Oncology |
| 1. Concepts 1 + 3 | telepsychology, telepsychiatry, telepsychotherapy, tele counselling |

**Table B Appraisal of sources, relevance, richness, rigour and author's theory of change**

| **First author (date)**  **Country** | **Relevance**  Author’s Theory of Change leading to intervention’s success | **Richness**  How might this contribute to theory building/testing***?*** | **Rigour**  Source credible?  Methods used appropriate? | **Findings**  Plausible?  Support other studies? |
| --- | --- | --- | --- | --- |
| Calton (2020)  USA | Professional is actively curious about patient’s environment, feelings and physical conditions and works with the constraints and possibilities of the video technology | Yes  Acceptability and interactivity reinforce NPT concepts | Yes, Author is an expert practitioner and assumed trustworthy source of advice. | Yes. But offers caution against telemedicine’s efficiency displacing emotional connection with patients and families. |
| Chávarri-Guerra (2021)  Mexico | Adaptation of existing coherent interventions is mobilised by a crisis. Refutes idea that success only happens in resource-rich contexts | Yes  Yes.  Refutes idea that success only happens in resource-rich contexts | Yes (Low/Moderate) Report doesn’t mention ethical approval. Survey results are descriptive statistics and qualitative comments. | Yes.  Of note: Identifies privacy issues, especially in the Mexican context of household overcrowding. |
| HospiceUK (2020)  UK | Videoconferencing successfully folded into usual care by attentive staff can enable best practice | Yes.  Imagery in blog and content give important contextual features. | Yes. Photograph of family features prominently. Optics convey equality, diversity and inclusion message. | Yes  Emotional support provided to patient and wife using video in the context of having a good inpatient experience. |
| HospiceUK (2022)  UK | Online interventions increase accessibility and reach of hospice care | Yes  Gives context for the review question, especially impact of CV-19 and under funding of hospice care  Yes.  For context and future strategy for cross-agency integration | Yes  Cites a variety of research and other publications connecting mental health and EOLC | Yes  Identifies benefits and challenges of online services. Lack of digital literacy, especially for older people, and financial poverty precludes access to digital services  Argues investment in EOLC will benefit people in work.  Addresses loneliness and isolation. |
| Keenan (2021)  Wales, UK | Discrepancies between Healthcare professionals’ and patients’ needs satisfaction explains why telehealth is not more routinely implemented | Yes  Intervention: psychosocial support (included art therapy) from qualified therapist on average once every ten days using the telehealth system  Yes.  Psychological needs of all involved as motivating behaviours and perceptions | Yes (Moderate)  Semi-structured interviews with professionals prior to the introduction of a telehealth service.  Patient data collected by semi-structured interviews at three-monthly intervals following the introduction of the service.  Deductive thematic analysis using Self-Determination Theory | Yes  Three themes identified that support other studies: autonomy, competence, and relatedness.  Convergence and divergence of perceptions between patients and professionals explored and differences highlighted.  Useful to get data from professionals ahead of implementation of telehealth |
| Mackey (2022)  USA | Video enabled collaboration and connection in situations of isolation | Yes  Emotional support during the COVID pandemic where “technology and tele-palliative care were utilized to reconnect the patient with clinicians and family and to provide clinical care that enhanced coping skills and support.”  Yes. confirms NPT concepts  Significance of social isolation | Yes, for a case description | Yes  illustrates the multidisciplinary team caring at the time of COVID and how technology was used to deliver emotional and spiritual care. Makes the point though that the advantages may only be limited to those with skills and tech resources. |
| Middleton-Green (2019)  England UK | Addresses experiences of  cross agency partnerships as motivators  Specialists as contextual factor | Yes.  Intervention aimed to reduce unplanned hospital admissions and improve out of hours care using video.  Service was meeting needs of patients not already known to specialist palliative care services. | Yes (High)  Analysis of referrals and calls to service April 2014-March 2015  Qualitative evaluation | Yes.  Agreed with others about helpfulness to service user of directly showing what was happening.  Being able to see staff was reassuring.  Evaluation highlighted some negative views, with some people wishing to control visual access to the home |
| Milbury (2020)  USA | No explanation for why video was means of delivery | Homogenous population of white, educated and wealthy. Incentivised | Yes (moderate/low)  Incentives:  Electronic follow-up assessments for which participants received $20 gift card  ($120 per couple in total) | Yes  Videoconference delivery was acceptable.  Significant effects for patients.  Dyadic design increased feasibility and retention. |
| Ozier (2019)  Canada | Videoconferencing intervention into patients’ homes enhances accessibility | Yes.  Contextual benefits and limitations to success | Yes (low/moderate)  standardised scales and psychometric tests completed weekly. Outcome measures pre and post intervention and 4 weeks later | Yes  Supports feasibility of intervention by video with patients with cognitive impairments due to brain tumours |
| Palma (2021)  Chile | Programme works in conditions of urgency and scarcity because staff adapt interventions | Yes  Yes.  Material and educational resources and cross disciplinary collaborations | Yes (moderate)  Descriptive statistics  User satisfaction measured through self-administered electronic survey | Yes.  Training provided. Inclusion of relatives in majority of sessions. Supports other studies that spiritual and psychological care possible by video. |
| Pearce (2021)  Ireland  Research | Delivery of intervention through video was an unwelcome outcome of the pandemic | Yes  Yes.  Unintended consequences of remote interventions | Yes (moderate) | Refutes positive narratives |
| Ritchey (2020)  USA | Widespread disruption created sense of urgency and vision for change mobilised collaborations | Yes.  Gives context about how service was transitioned to video in response to COVID-19 pandemic.  Illustrates with a case how success was evaluated of the PC telehealth program  Yes. Describes the actions and motivations of people collaborating. Relationality of palliative care preserved | Yes (moderate)  Uses business model to evaluate successful use of video telehealth for palliative care | Yes  Confirms pandemic as motivator for system change, with positive outcomes. But sees “potential for telehealth visits near the EOL to go terribly wrong” |
| Stern (2012)  Canada | Offers two contextual factors:  enhanced access to care and usability of the home telehealth system. | Yes. Focus on the usage of home telehealth where emotional support was reported as one example. | Yes (Moderate)  Quantitative data from electronic records for patterns of home telehealth. Qualitative data: interviews, direct observation, and nursing documentation | Agrees with acceptability of video use, and families’ experience of video as reassuring.  Negative consequences of telehealth highlighted lack of integration of services, inappropriate  timing of the intervention and  technical problems |
